# Experimental system enables studies of *Mycobacterium tuberculosis* during aerogenic transmission

**DOI:** 10.1128/mbio.00958-25

**Published:** 2025-08-25

**Authors:** Frank Nuritdinov, Joshua Woo, Markus J. Schmidt, Narineh M. Odjourian, Melissa Cristaldo, Maureen Dougher, Rosleine Antilus-Sainte, Thomas Heldt, Kyu Rhee, Lydia Bourouiba, Martin Gengenbacher

**Affiliations:** 1 Center for Discovery and Innovation, Hackensack Meridian Health, Nutley, New Jersey, USA; 2 Fluids and Health Network, Institute for Medical Engineering and Science, Massachusetts Institute of Technology 2167, Cambridge, Massachusetts, USA; 3 Division of Infectious Diseases, Weill Department of Medicine, Weill Cornell Medical College 12295 , New York, New York, USA; 4 Hackensack Meridian School of Medicine 576909 , Nutley, New Jersey, USA; Washington University in St. Louis School of Medicine, St. Louis, Missouri, USA

**Keywords:** TB, simulated transmission, aerosol infection, particle size distribution, mouse model

## Abstract

Tuberculosis, a persistent public health challenge worldwide, is transmitted when exhaled *Mycobacterium tuberculosis* (Mtb) particles expelled from an infected individual are inhaled by a susceptible person. To study the adaptation of Mtb during transition between hosts, we developed a transmission simulation system (TSS) that combines controlled pathogen aerosolization and measurement of bioaerosol particle characteristics with in-flight sampling of Mtb and infection of mice by nose-only exposure. Using scattered-light spectrometry, we demonstrated that Mtb aerosol concentrations generated by the TSS better represented human cough than the aerosol concentrations produced by a full-body inhalation exposure system commonly used for Mtb infection of mice. Additionally, the TSS deposited clinically relevant low doses of Mtb into murine lungs with greater precision than the full-body inhalation exposure systems. The TSS revealed a linear correlation between Mtb inoculum concentration and pathogen deposition in murine lungs up to 200 colony-forming units. Higher inoculum concentrations led to a reduction in total particle number, which resulted in disproportionately lower pulmonary infection doses. Importantly, the particle size distributions of Mtb-laden aerosols produced by the TSS mirrored those of tuberculosis patient coughs, with 90% of culturable Mtb found in particles with aerodynamic diameters below 3.3 µm. In conclusion, the TSS represents a novel effective and precise translational platform enabling detailed biophysical and molecular studies of Mtb transmission.

**IMPORTANCE:**

Tuberculosis is transmitted when exhaled *Mycobacterium tuberculosis* (Mtb)-laden microdroplets of an infected individual are inhaled by a susceptible person. Historically, studies on Mtb transmission have focused mainly on epidemiology due to the technical challenges in replicating the transmission process effectively in a laboratory setting. In this study, we introduce a transmission simulation system (TSS) that integrates controlled Mtb aerosolization, biophysical aerosol particle measurements, in-flight Mtb sampling, and aerosol infection of mice. The TSS generated Mtb bioaerosol concentrations comparable to those produced by human coughs. These pathogen droplets were accurately deposited in mouse lungs at low Mtb doses relevant to human transmission. Notably, the distribution of Mtb among aerosol particles of various sizes closely mirrored that found in the coughs of tuberculosis patients. In summary, the TSS represents a novel tool for conducting molecular studies of Mtb transmission through the air.

## OBSERVATION

Tuberculosis (TB) is an airborne disease that remains the leading cause of death from a single infectious pathogen (1). TB transmission occurs through the air when the exhaled *Mycobacterium tuberculosis* (Mtb)-laden bioaerosols of an infected individual are inhaled by a susceptible person and deposited in their lungs (2). Due to technical challenges in characterizing and controlling physiologically relevant pathogen-laden microdroplets to replicate this complex process faithfully in a laboratory setting, past TB transmission studies were largely epidemiology-centered (2–6). Recently, an *in vitro* approach designed to replicate the conditions Mtb encounters during its journey between hosts revealed genes that are regulated throughout this process (7). However, a preclinical animal model to validate and further study the candidate transmission survival genome is lacking.

To close this critical gap, we developed a transmission simulation system (TSS) consisting of a 36-port rodent nose-only inhalation system with a single-jet Blaustein atomizer module (CH Technologies) (8), fed by a syringe pump at 12 mL/h with mid-log Mtb H37Rv (ATCC#27294) in phosphate-buffered saline (PBS) and a dilution air 1/2 flow rate of 3 L/min. Additional features of the TSS included an impinger for Mtb aerosol sampling, a scattered-light aerosol spectrometer (Promo 2000 with Aerosol Sensor welas 2070, Palas) for biophysical particle characterization and a six-stage viable Andersen cascade impactor (Tisch Environmental) (9) to measure the viable Mtb-laden aerosol size distribution. The final TSS design and the settings used in our experiments were the result of comprehensive characterization (with particles rather than pathogens and with fluids recapitulating various physiological fluid properties) aimed at generating bioaerosol particles that are compatible with a range of prior measurements of bioaerosols from human cough (4, 10). For reference, our studies included the full-body inhalation exposure system (FES) (Glas-col), which is widely used to inoculate mice in Mtb research across preclinical models (11, 12). All procedures involving animals were reviewed and approved by the Hackensack Meridian Health institutional animal care and use committee and used 10-week-old BALB/c mice (Charles River Laboratories).

**Fig 1:**
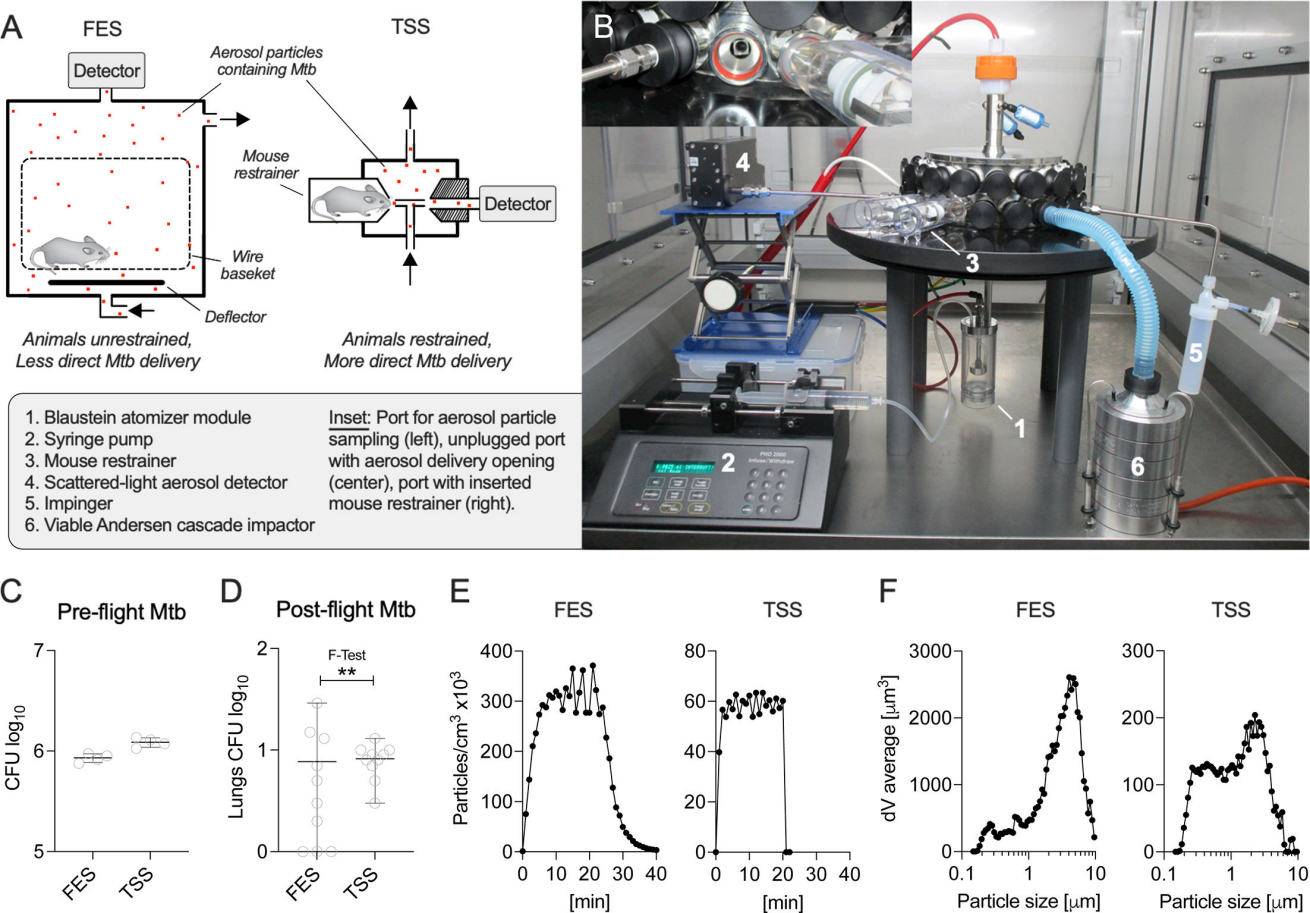
The TSS achieves low doses of Mtb lung infection of mice more consistently than the FES. (A) Schematic representation of the FES (full-body) and TSS (nose-only). Arrows indicate the flow path of aerosol particles (red dots). (B) Components of the TSS. (C) Mice in a wire-mesh baskets (FES) or in restrainer tubes attached to infection ports (TSS) were exposed to aerosolized Mtb for ~20 min. The total colony-forming unit (CFU)s of Mtb aerosolized during the entire infection cycle (*n* = 4). (D) At 24 h postinfection, mice were euthanized to determine the Mtb load in lungs (*n* = 10) by plating organ homogenate on agar. The F-test was performed to compare variances between study groups. Particle concentration represented as average over 1-min intervals (E) and particle volume size distribution (F) was measured online by scattered-light aerosol spectrometry. **P < 0.01. One out of two biological replicates is shown.

The designs of the FES and TSS systems differ significantly (Fig. 1A and B). The FES utilizes a Venturi nebulizer that recirculates a fixed 5-mL inoculum, whereas the TSS uses a single-jet Blaustein atomizer with a continuous supply of fresh inoculum. In the FES, the entire 5-mL inoculum is aerosolized over approximately 20 min. For comparability, all TSS experiments employed a consistent infection period of 20 min, during which 4 mL of inoculum was aerosolized. To account for the difference in inoculum volume, the Mtb culture densities were adjusted to aerosolize approximately 10^6^ CFU of Mtb over the infection cycle in both systems (Fig. 1C). Under these conditions, the FES and TSS implanted 7.7 ± 8.6 CFU and 8.2 ± 2.6 CFU into the lungs of mice, respectively, as determined by plating the entire homogenized organ in PBS with 0.05% Tween 80 on Middlebrook 7H11 agar (Fig. 1D) (13). At this infection dose, the FES and TSS resulted in infection rates of 70% and 100%, respectively, with the TSS cohort showing significantly less variance (Fig. 1D). These findings align with those of Plumlee and colleagues, who reported similar infection rates for the FES at very low Mtb doses (14), highlighting the superior precision of nose-only Mtb-aerosol delivery (TSS) compared with whole-body exposure (FES).

For biophysical characterization, aerosols were sampled from the top of the FES infection chamber, while the detector assembly was inserted into one of the 36 mouse restrainer ports of the TSS, allowing precise characterization of aerosol particles delivered to the nose of an individual animal (Fig. 1A). Due to its larger infection chamber, the FES required 8 min to reach the maximum aerosol concentration of 315,836 ± 31,084 particles/cm³, whereas the TSS achieved its peak output of 58,617 ± 3,224 particles/cm³ after 2 min (Fig. 1E). Compared with the FES, the aerosol concentration produced by the TSS more closely approximated the range observed in human cough (up to 5,000 particles/cm³) (4, 10). The FES generated a higher proportion of large particles, with larger aerodynamic diameter than the TSS (Fig. 1F). Smaller microdroplets have a higher likelihood to deposit in anatomically relevant locations of the lungs, particularly for the smaller animal models used here (5), making them more relevant for transmission by inhalation (15). Indeed, such size range of microdroplets desiccate partially or in full, over a fraction of second to seconds, depending on how they are exhaled and can remain airborne for extended time, posing high risk of transmission via inhalation (4, 16).

**Fig 2:**
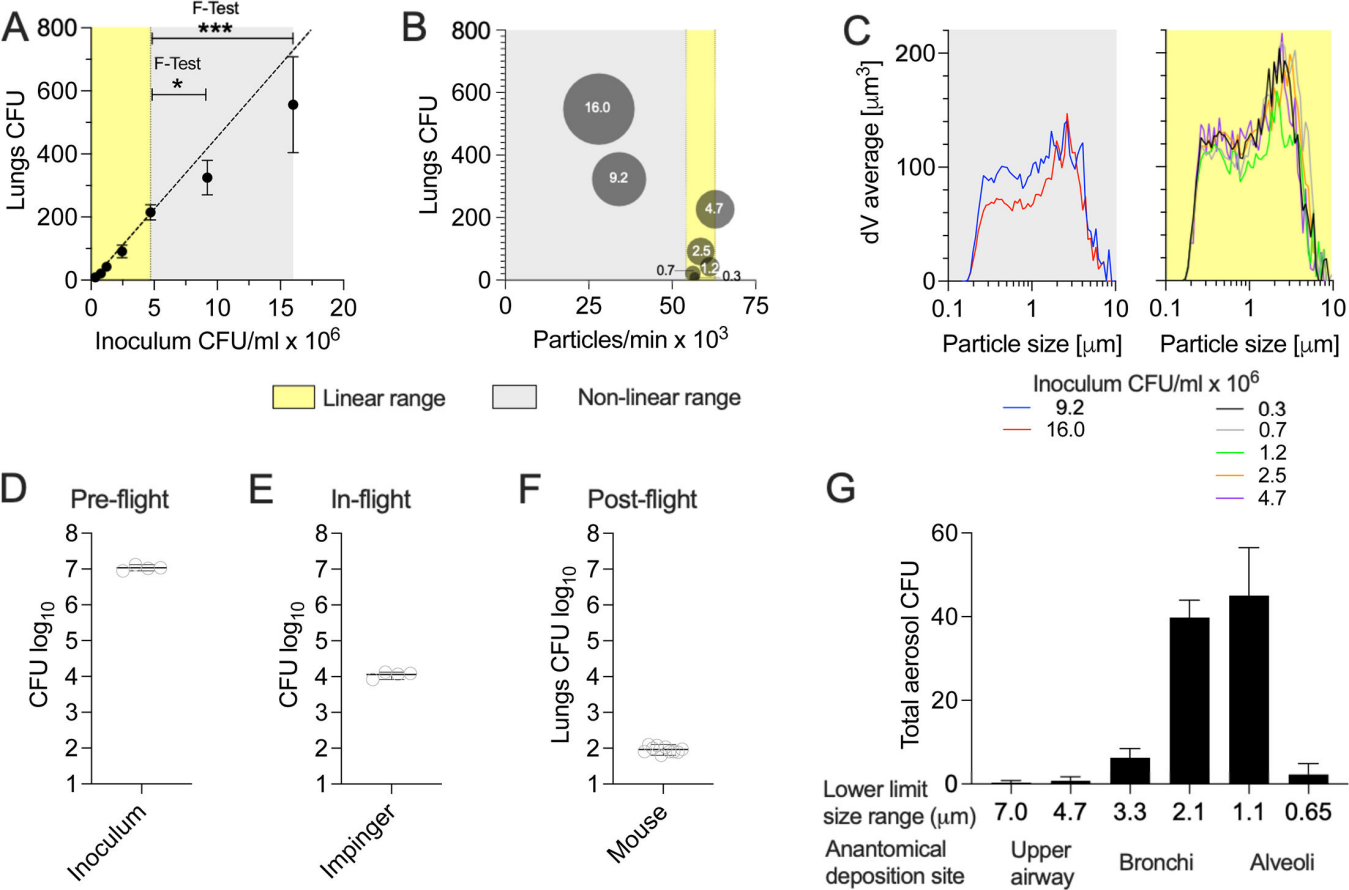
The TSS generates Mtb aerosols with a size distribution similar to the cough of active TB patients. (A) Groups of 10 mice were inoculated with Mtb suspensions at specified concentrations for 20 min using the TSS. At 24 h postinfection, the bacterial load of lungs was determined by plating lung homogenates on agar. Linear regression (dashed line) was performed excluding the two largest inoculums. The F-test was used to compare variances between study groups. (B) Relation of inoculum Mtb concentration (numbers in blobs × 10^6^ CFU), aerosol particles/min delivered to individual mice (*x*-axis) and Mtb deposited in lungs (*y*-axis). (C) Particle size distribution during infection with the two largest inoculums (left) and five smallest inoculums (right). The specified inoculum (D) was aerosolized within 20 min, and an impinger was used to collect total Mtb aerosols delivered to a single rodent port (E) while 10 mice were infected. (F) The pathogen burden in mouse lungs was determined at 24 h postinfection by plating organ homogenates on agar. (G) After mice were infected, a viable Andersen cascade impactor was connected to the TSS to determine the Mtb particle size (aerodynamic diameter) distribution. Data are represented as mean and SD. *P < 0.05, ***P < 0.001. One representative data set out of two biological replicates is shown.

Next, we assessed the dynamic range of the TSS by infecting cohorts of mice with Mtb serially diluted in PBS and measuring bacterial load in the lungs 24 h postinfection. The CFU in organ homogenates and infection inoculums was determined by plating samples serially diluted in PBS/Tween 80 onto Middlebrook 7H11 agar prior to outgrowth. A linear correlation was observed up to 200 CFU implanted in the lungs and Mtb concentrations ranging from 0.3 to 4.7 × 10^6^ CFU/mL (Fig. 2A). However, at concentrations of 9.2 and 16.0 × 10^6^ CFU/mL, we found significantly higher variances in lung deposition, with the average number of Mtb recovered falling below the linear projection (Fig. 2A). Biophysical characterization of aerosol particles during mouse infection showed that aerosolizing inoculums with up to 4.7 × 10^6^ CFU/mL delivered 54-63 × 10^3^ particles/min to individual animals. In contrast, higher Mtb concentrations resulted in significantly lower aerosol particle concentrations (Fig. 2B). The reduction in particle concentration with larger inoculums was evident across all particle sizes, as reflected in the decrease of all average particle volumes, though the shape of the volume histograms was preserved (Fig. 2C). Lipophilic Mtb bacilli form biofilm-like cords, which contribute to their immunopathology and drug tolerance (17, 18). To prevent aggregate formation in Mtb cultures, detergents are often added to the growth media (19). However, we chose not to use detergents in our study, as they alter the cell wall composition and reduce virulence (20, 21). Consequently, the lipophilic interactions of Mtb may have impacted the fluid fragmentation process of the TSS at higher pathogen concentrations.

To demonstrate the capabilities of the TSS, we selected an inoculum within the linear range that delivers approximately 100 CFU to the mouse lungs during a 20-min infection cycle (Fig. 2A). Over the course of this period, a total of 1.08 × 10^7^ CFU were aerosolized (Fig. 2D). Using an impinger device (Fig. 1B) inserted into one of the 36 rodent ports, we captured 1.14 × 10^4^ CFU, which represents the total number of viable Mtb delivered to a single animal (Fig. 2E). The impinger collected infectious aerosols in 4 mL of Middlebrook 7H9 broth, which were then plated on agar for CFU enumeration. Of the total Mtb delivered to each mouse, an average of 93 ± 19 CFU (~1%) successfully implanted in the lungs via tidal breathing (Fig. 2F). We utilized a viable Andersen cascade impactor (Fig. 1B), specifically designed to sort viable pathogen-laden particles according to their aerodynamic diameter and direct them onto an agar surface for subsequent outgrowth (9). The various stages of the cascade (i.e., particle size bins) represent distinct anatomical deposition sites in the lungs (22). In our experiment, 90% of the viable Mtb particles had sizes below aerodynamic diameters of 3.3 µm (Fig. 2G) capable of reaching the smaller bronchi and alveoli in the lungs. Interestingly, the overall distribution of Mtb particle sizes produced by the TSS closely resembled those reported for coughs of patients with active TB (15, 23).

In summary, the TSS facilitated precise and efficient infection of mice with Mtb-laden aerosols relevant to human TB transmission. Given the critical importance of TB control, the TSS is a translational platform for developing and testing novel transmission intervention strategies.

## References

[1] WHO. 2024. Global tuberculosis report 2024. World Health Organization, Geneva.

[2] Churchyard G, Kim P, Shah NS, Rustomjee R, Gandhi N, Mathema B, Dowdy D, Kasmar A, Cardenas V. 2017. what we know about tuberculosis transmission: an overview. J Infect Dis 216:S629–S635. doi:10.1093/infdis/jix362 29112747 PMC5791742

[3] Martinez L, Shen Y, Mupere E, Kizza A, Hill PC, Whalen CC. 2017. Transmission of Mycobacterium tuberculosis in households and the community: a systematic review and meta-analysis. Am J Epidemiol 185:1327–1339. doi:10.1093/aje/kwx025 28982226 PMC6248487

[4] Bourouiba L. 2021. Fluid dynamics of respiratory infectious diseases. Annu Rev Biomed Eng 23:547–577. doi:10.1146/annurev-bioeng-111820-025044 34255991

[5] WHO. 2024. Global technical consultation report on proposed terminology for pathogens that transmit through the air. World Health Organization, Geneva

[6] Alsved M, Bourouiba L, Duchaine C, Löndahl J, Marr LC, Parker ST, Prussin AJ II, Thomas RJ. 2020. Natural sources and experimental generation of bioaerosols: challenges and perspectives. Aerosol Sci Technol 54:547–571. doi:10.1080/02786826.2019.1682509

[7] Mishra S, Singh PR, Hu X, Lopez-Quezada L, Jinich A, Jahn R, Geurts L, Shen N, DeJesus MA, Hartman T, Rhee K, Zimmerman M, Dartois V, Jones RM, Jiang X, Almada-Monter R, Bourouiba L, Nathan C. 2025. Candidate transmission survival genome of Mycobacterium tuberculosis . Proc Natl Acad Sci USA 122:e2425981122. doi:10.1073/pnas.2425981122 40053362 PMC11912377

[8] Alfarra R, Baltensperger U, Bell D, Danelli S, Biagio C, Doussin J-F, Formenti P, Gysel M, Massabò D, McFiggans G, Modini R, Möhler O, Prati P, Saathoff H, Wenger J. 2023. Preparation of the experiment: addition of particles, p 163–206. In HF Jean-François Doussin, Kiendler-Scharr Astrid, Seakins Paul, Wenger John (ed), A practical guide to atmospheric simulation chambers. Springer.

[9] Andersen AA. 1958. New sampler for the collection, sizing, and enumeration of viable airborne particles. J Bacteriol 76:471–484. doi:10.1128/jb.76.5.471-484.1958 13598704 PMC290224

[10] Yang S, Lee GWM, Chen C-M, Wu C-C, Yu K-P. 2007. The size and concentration of droplets generated by coughing in human subjects. J Aerosol Med 20:484–494. doi:10.1089/jam.2007.0610 18158720

[11] Gengenbacher M, Zimmerman MD, Sarathy JP, Kaya F, Wang H, Mina M, Carter C, Hossen MA, Su H, Trujillo C, Ehrt S, Schnappinger D, Dartois V. 2020. Tissue distribution of doxycycline in animal models of tuberculosis. Antimicrob Agents Chemother 64:e02479-19. doi:10.1128/AAC.02479-19 32041718 PMC7179585

[12] Xie M, Tsai CY, Woo J, Nuritdinov F, Cristaldo M, Odjourian NM, Antilus-Sainte R, Dougher M, Gengenbacher M. 2025. BAFF and APRIL immunotherapy following bacille calmette-guérin vaccination enhances protection against pulmonary tuberculosis in mice. Front Immunol 16:1551183. doi:10.3389/fimmu.2025.1551183 39981256 PMC11839638

[13] Dick T, Shin SJ, Koh WJ, Dartois V, Gengenbacher M. 2020. Rifabutin is active against Mycobacterium abscessus in mice. Antimicrob Agents Chemother 64:e01943-19. doi:10.1128/AAC.01943-19 31767722 PMC6985736

[14] Plumlee CR, Duffy FJ, Gern BH, Delahaye JL, Cohen SB, Stoltzfus CR, Rustad TR, Hansen SG, Axthelm MK, Picker LJ, Aitchison JD, Sherman DR, Ganusov VV, Gerner MY, Zak DE, Urdahl KB. 2021. Ultra-low dose aerosol infection of mice with Mycobacterium tuberculosis more closely models human tuberculosis. Cell Host Microbe 29:68–82. doi:10.1016/j.chom.2020.10.003 33142108 PMC7854984

[15] Fennelly KP, Jones-López EC, Ayakaka I, Kim S, Menyha H, Kirenga B, Muchwa C, Joloba M, Dryden-Peterson S, Reilly N, Okwera A, Elliott AM, Smith PG, Mugerwa RD, Eisenach KD, Ellner JJ. 2012. Variability of infectious aerosols produced during coughing by patients with pulmonary tuberculosis. Am J Respir Crit Care Med 186:450–457. doi:10.1164/rccm.201203-0444OC 22798319 PMC3443801

[16] Fernstrom A, Goldblatt M. 2013. Aerobiology and its role in the transmission of infectious diseases. J Pathog 2013:493960. doi:10.1155/2013/493960 23365758 PMC3556854

[17] Mishra R, Hannebelle M, Patil VP, Dubois A, Garcia-Mouton C, Kirsch GM, Jan M, Sharma K, Guex N, Sordet-Dessimoz J, Perez-Gil J, Prakash M, Knott GW, Dhar N, McKinney JD, Thacker VV. 2023. Mechanopathology of biofilm-like Mycobacterium tuberculosis cords. Cell 186:5135–5150. doi:10.1016/j.cell.2023.09.016 37865090 PMC10642369

[18] Du Toit A. 2023. Mycobacterial cords. Nat Rev Microbiol 21:769–769. doi:10.1038/s41579-023-00989-w 37875606

[19] Wallace E, Hendrickson D, Tolli N, Mehaffy C, Pena M, Nick JA, Knabenbaur P, Watkins J, Simpson A, Amin AG, Chatterjee D, Dobos KM, Lahiri R, Adams L, Strong M, Salfinger M, Bradford R, Stedman TT, Riojas MA, Hazbon MH. 2021. Culturing Mycobacteria. Methods Mol Biol 2314:1–58. doi:10.1007/978-1-0716-1460-0_1 34235647

[20] Ortalo-Magné A, Lemassu A, Lanéelle MA, Bardou F, Silve G, Gounon P, Marchal G, Daffé M. 1996. Identification of the surface-exposed lipids on the cell envelopes of Mycobacterium tuberculosis and other mycobacterial species. J Bacteriol 178:456–461. doi:10.1128/jb.178.2.456-461.1996 8550466 PMC177678

[21] Leisching G, Pietersen RD, Wiid I, Baker B. 2016. Virulence, biochemistry, morphology and host-interacting properties of detergent-free cultured mycobacteria: an update. Tuberculosis (Edinb) 100:53–60. doi:10.1016/j.tube.2016.07.002 27553410

[22] Carvalho TC, Peters JI, Williams RO. 2011. Influence of particle size on regional lung deposition--what evidence is there? Int J Pharm 406:1–10. doi:10.1016/j.ijpharm.2010.12.040 21232585

[23] Fennelly KP. 2020. Particle sizes of infectious aerosols: implications for infection control. Lancet Respir Med 8:914–924. doi:10.1016/S2213-2600(20)30323-4 32717211 PMC7380927

